# Insomnia symptoms and risk of cardiovascular diseases among 0.5 million adults: A 10-year cohort

**DOI:** 10.1212/WNL.0000000000008581

**Published:** 2019-12-03

**Authors:** Bang Zheng, Canqing Yu, Jun Lv, Yu Guo, Zheng Bian, Mi Zhou, Ling Yang, Yiping Chen, Xiaojun Li, Ju Zou, Feng Ning, Junshi Chen, Zhengming Chen, Liming Li, on behalf of the China Kadoorie Biobank Collaborative Group

**Affiliations:** From the Department of Epidemiology and Biostatistics (B.Z., C.Y., J.L., M.Z., L.L.), School of Public Health, Peking University Health Science Center, Beijing, China; Neuroepidemiology and Aging Research Unit (B.Z.), School of Public Health, Imperial College London, UK; Chinese Academy of Medical Sciences (Y.G., Z.B., L.L.), Beijing; Clinical Trial Service Unit & Epidemiological Studies Unit (L.Y., Y.C., Z.C.), Nuffield Department of Population Health, University of Oxford, UK; Jili Community Health Service (X.L., J.Z.), Liuyang, Hunan; Qingdao Center for Disease Control and Prevention (F.N.), Shandong; and China National Center for Food Safety Risk Assessment (J.C.), Beijing.

## Abstract

**Objective:**

To examine the associations of individual insomnia symptoms with risks of incident cardio-cerebral vascular diseases (CVD) and possible moderating factors among Chinese adults.

**Methods:**

The China Kadoorie Biobank is a prospective cohort study that recruited participants from 10 areas across China. Data from 487,200 adults 30 to 79 years of age who were free of stroke, coronary heart disease, and cancer at baseline were analyzed. Three insomnia symptoms were assessed with self-reported difficulties in initiating or maintaining sleep, early morning awakening, and daytime dysfunction for at least 3 d/wk at baseline. Incidences of CVD were followed up through disease registries and national health insurance databases until 2016.

**Results:**

During a median of 9.6 years of follow-up, 130,032 cases of CVD were documented. Cox regressions showed that 3 insomnia symptoms were associated with increased risk of total CVD, with respective adjusted hazard ratios (HRs) and 95% confidence intervals (CIs) of 1.09 (95% CI 1.07–1.11), 1.07 (95% CI 1.05–1.09), and 1.13 (95% CI 1.09–1.18). Participants with individual symptoms also had higher risks of ischemic heart disease (IHD; HR 1.13, 1.09, and 1.17) and ischemic stroke but not hemorrhagic stroke. Participants with all 3 symptoms were at an 18%, 22%, or 10% higher risk of CVD, IHD, or ischemic stroke compared to nonsymptomatic adults. Associations between 3 symptoms and CVD incidence were consistently stronger in younger adults or those without baseline hypertension (*p* for interaction <0.05).

**Conclusions:**

Individual and coexisting insomnia symptoms are independent risk factors for CVD incidence, especially among young adults or adults who have not developed hypertension.


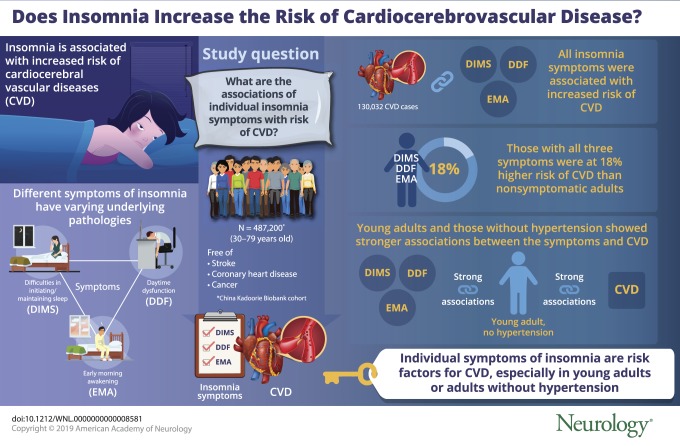


Insomnia, characterized by poor sleep quality of having difficulties in initiating or maintaining sleep (DIMS) and often accompanied by daytime dysfunction (DDF), is the commonest sleep disorder and the second most prevalent mental disorder throughout the world.^1–3^ According to the China Chronic Disease and Risk Factor Surveillance Study, 35.7% of Chinese residents 15 to 69 years of age reported poor sleep quality.^4^ Previous studies have shown that insomnia has adverse effects on physical health and mental health in the general population.^5^

Emerging evidence from prospective studies suggested that insomnia disorder is associated with increased risks of cardio-cerebral vascular diseases (CVD).^6–8^ A recent meta-analysis of 17 cohort studies indicated that insomnia significantly increased CVD mortality (hazard ratio [HR] 1.33, 95% confidence interval [CI] 1.13–1.57) and risks of individual types of CVD, including myocardial infarction (MI), coronary heart disease (CHD), and stroke.^9^

However, the definitions of insomnia in most previous cohort studies are not clear enough or are simply based on the symptom of having difficulties initiating sleep,^9,10^ while other insomnia symptoms have not been fully investigated. Compared with clinically diagnosed insomnia meeting the frequency, duration, and severity criteria, individual insomnia symptoms are easier to assess in large-scale studies and population screenings and thus have more public health significance. Because different insomnia symptoms are related to distinct underlying mechanisms and pathologic changes (e.g., early morning awakening [EMA] reflects circadian rhythmic disruption rather than a delay of the circadian cycle phase),^11,12^ it is possible that their effects on CVD risks could vary dramatically. Moreover, few studies have explored the associations between coexisting insomnia symptoms and CVD incidences. There is also limited and controversial evidence regarding whether the insomnia-CVD associations vary by age, sex, or other population characteristics.^7,8,13^ Therefore, to comprehensively clarify the relationships between individual insomnia symptoms and CVD incidence and potential effect modifiers, this study was conducted using the large-scale cohort data from the China Kadoorie Biobank (CKB) Study.^14^

## Methods

### Study population

The CKB Study is an ongoing population-based prospective study that aims to assess the complex interplay of lifestyle, environmental, and genetic factors as determinants of chronic diseases.^14^ Details of this study have been previously reported.^15^ Briefly, the CKB cohort included 512,715 adults (age 30–79 years) from 10 geographically diverse areas across China who completed a baseline survey during 2004 to 2008 and have been followed up since.

In the present study, individuals were excluded from analyses if they had a history of stroke (n = 8,884), CHD (n = 15,472), or cancer (n = 2,578) at baseline. After exclusions, 487,200 participants (199,241 men and 287,959 women) remained in the analyses.

### Assessment of insomnia symptoms

The baseline survey of CKB study included questions pertaining to specific insomnia symptoms for at least 3 d/wk in the past month, the frequency threshold of which was in compliance with established clinical diagnostic criteria.^16^ Subjects were classified as having DIMS if they reported having trouble falling asleep (sleep onset latency ≥30 minutes) after going to bed or waking up in the middle of the night; those who reported waking up too early and not be able to get back to sleep were classified as having EMA; those who reported having trouble keeping sober-minded during daytime because of bad sleep were classified as having DDF; and those who reported 1 or more of the 3 symptoms were classified as having any insomnia symptoms.

Information on covariates was obtained from the baseline survey and physical examination,^14,15^ including sociodemographic characteristics, lifestyle factors, body mass index (BMI; weight in kilograms divided by height in meters squared), medical history, other sleep variables, and mental health status.

### Ascertainment of CVD outcomes

Information on CVD incidence was collected through linkages with established disease registries, the new national health insurance claim databases, and local residential records.^15^ Electronic linkage to the national health insurance databases has covered 95% of the participants in 2013 and has been renewed annually. To minimize underreporting of diseases and to identify those who were lost to follow-up, participants not linked to the health insurance databases were actively followed up annually by staff to ascertain their status of hospital admission, death, or moving out of the study area.

Trained staff blinded to baseline information of participants coded all diagnoses and deaths that occurred during follow-up using the ICD-10. The main outcomes of interest included the incidences of CVD (I00–I99); ischemic heart disease (IHD, I20–I25) and acute MI (I21); and stroke (I60–I61; I63-I64), hemorrhagic stroke (I61), and ischemic stroke (I63).

### Statistical analysis

Descriptive analyses were conducted to compare the distributions of baseline characteristics among participants with and without specific insomnia symptoms. The differences of means or proportions of baseline variables between groups were estimated with general linear regression or binary/multinomial logistic regression models, respectively, controlling for age, sex, and study areas. Follow-up time of each participant was defined as the time interval from the baseline survey (2004–2008) to the diagnosis of the specific outcome, death resulting from any cause, loss to follow-up, or December 31, 2016, whichever occurred first. Cox proportional hazard models were used to estimate the HRs and 95% CIs for the associations between each of the 3 insomnia symptoms (DIMS, EMA, DDF) and CVD outcomes, with age as the underlying time scale. To account for the group-specific effect, the models were stratified jointly by sex, 10 study areas, and age at baseline in 5-year intervals.

The Cox regression models were adjusted for established and potential confounding factors sequentially. We included age at baseline (continuous) in the first model and additionally adjusted for the following covariates in the second model: level of education (no formal school, primary school, middle school, high school, college, or university or higher); annual household income (<2,500, 2,500–4,999, 5,000–9,999, 10,000–19,999, 20,000–34,999, or ≥35,000 RMB); marital status (married, widowed, divorced or separated, or never married); alcohol consumption (not weekly drinking, former regular drinker, weekly but not daily drinking, daily drinker of <15, 15–29, 30–59, or ≥60 g/d); smoking status (never/occasional smoker, former smoker, regular smoker of 1–14, 15–24, or ≥25 cigarettes/d); tea consumption (never/almost never, occasionally, only at certain seasons, every month but less than weekly, or usually at least once a week); physical activity level in metabolic equivalent of task–hours per day (continuous); intake frequencies of red meat, fresh fruits, and fresh vegetables (daily, 4–6 d/wk, 1–3 d/wk, monthly, or rarely/never); and family history of heart attack and stroke (presence or absence). We further adjusted for possible mediators/confounders in the third model, including BMI (<24.0, 24.0–27.9, ≥28.0 kg/m^2^), prevalent hypertension and diabetes mellitus at baseline (presence or absence), frequent use of sleep aid medications (frequent users or not), frequency of snoring during sleep (frequently, sometimes, never/do not know), and mental health status (having depression or anxiety symptoms or not). The interaction terms between different insomnia symptoms were then tested in the full model. Sex-specific modeling was also conducted to examine the possible sex heterogeneity in the insomnia-CVD associations. Furthermore, we estimated the HRs of CVD incidences by the number of insomnia symptoms (taking participants with no symptoms as the reference group) and any insomnia symptoms following the same modeling procedures. Tests for linear trend were conducted by modeling the number of insomnia symptoms as a continuous variable.

To examine the robustness of the main findings, we also conducted several sensitivity analyses: additionally adjusting for the 3 insomnia symptoms mutually to estimate their associations with CVD risks independently of each other; additionally adjusting for occupation, household size, menopausal status (for women only; premenopausal, perimenopausal, or postmenopausal), history of chronic obstructive pulmonary disease (presence or absence), diseases causing chronic pain (peptic ulcer, gallstone/gallbladder disease, rheumatoid arthritis), and psychiatric disorders; excluding frequent users of sleep aid medication; and excluding participants developing or dying of CVDs during the first 2 years of follow-up to rule out the possibility of reverse causality.

Subgroup analyses were further conducted to examine possible moderating effects of baseline characteristics on the insomnia-CVD associations. We estimated the associations among prespecified baseline subgroups based on age (<50, 50–59, or ≥60 years), BMI (<24.0, 24.0–27.9, or ≥28.0 kg/m^2^), level of physical activity (categorized with tertile cutoffs), prevalent hypertension and diabetes mellitus at baseline (presence or absence), and sleep duration (<7, 7–9, or >9 hours, based on the National Sleep Foundation's recommendations^17^). Effect modifications by these variables were tested by comparing the models with and without corresponding interaction terms using likelihood ratio tests.

Statistical analyses were performed with Stata (version 14, StataCorp, College Station, TX). All statistical tests were 2 sided, and the statistical significance was defined as *p* < 0.05.

### Standard protocol approvals, registrations, and patient consents

The study was approved by the Ethical Review Committee of the Chinese Center for Disease Control and Prevention (Beijing, China) and the Oxford Tropical Research Ethics Committee, University of Oxford (UK). All study participants provided written informed consent.

### Data availability

Cohort description and questionnaires are available at p3gobservatory.org/questionnaire/list.htm. Statistical code is available from Dr. Yu (e-mail, yucanqing@pku.edu.cn). For the data set, please refer to the CKB Study website (ckbiobank.org) for data access policy and procedures.

## Results

### Baseline characteristics of participants by insomnia symptoms

Among the 487,200 participants included in the analyses, 11.3% reported having the symptom of DIMS, 10.4% reported having EMA, and 2.2% reported having DDF. Compared with participants without specific insomnia symptoms, those with DIMS, EMA, or DDF were older, more likely to be female, not married, and from rural area; had lower education level, household income, and BMI; were more likely to report a history of diabetes mellitus; and had depression or anxiety symptoms (*p* < 0.05, table 1).

**Table 1:**
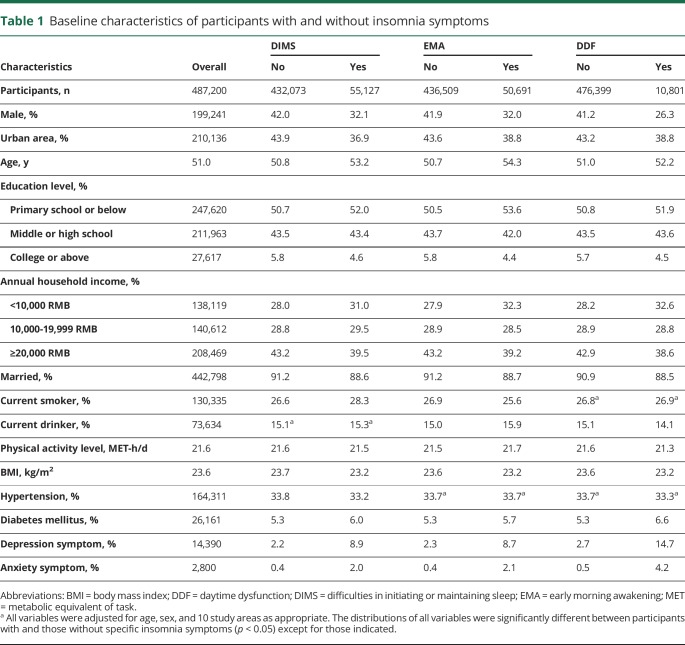
Baseline characteristics of participants with and without insomnia symptoms

### Associations of insomnia symptoms with total and specific types of CVD incidence

During a median follow-up of 9.6 years (4,317,504 total person-years), 130,032 cases of total CVD incidence, 40,348 cases of IHD, and 45,316 cases of stroke incidence were documented. After full adjustment for potential confounders, the insomnia symptoms of DIMS, EMA, and DDF were all associated with increased risks of total CVD incidence, and the respective HRs (95% CIs) were 1.09 (1.07–1.11), 1.07 (1.05–1.09), and 1.13 (1.09–1.18) (table 2).

**Table 2:**
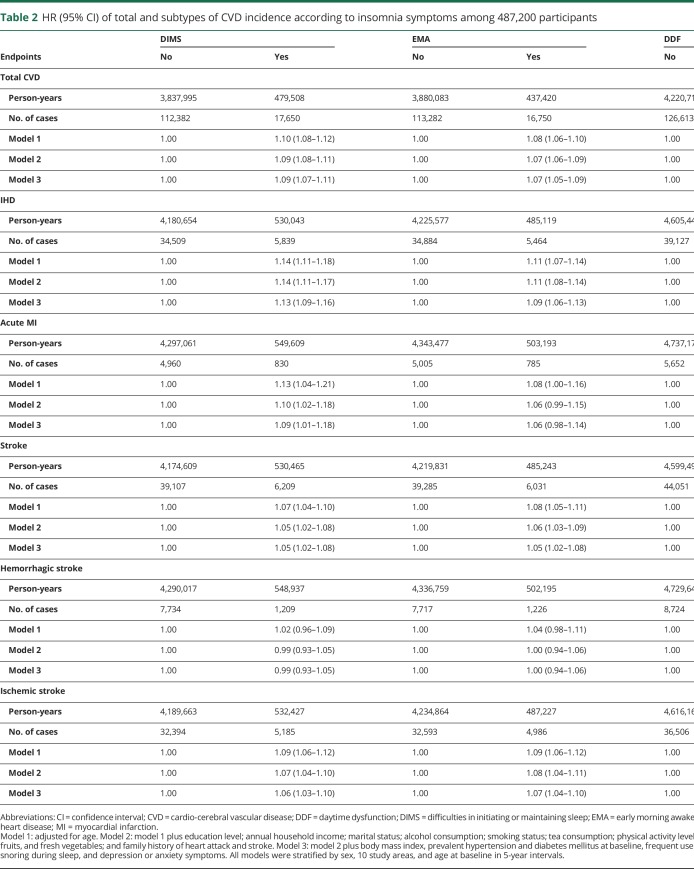

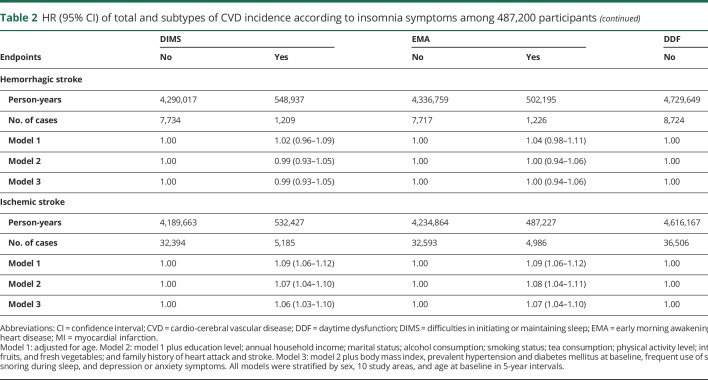
HR (95% CI) of total and subtypes of CVD incidence according to insomnia symptoms among 487,200 participants

Participants with DIMS, EMA, or DDF also had higher risks of IHD incidence compared with those without corresponding symptoms (HR 1.13, 1.09, and 1.17, *p* < 0.05). Only DIMS was associated with higher risk of acute MI (HR 1.09, *p* < 0.05). All 3 symptoms were associated with slightly increased risks of total stroke incidence (HR 1.05, 1.05, and 1.08) and ischemic stroke incidence (HR 1.06, 1.07, and 1.09, *p* < 0.05). No associations were observed between the 3 symptoms and hemorrhagic stroke incidence (*p* > 0.05, table 2). No interactions between insomnia symptoms on CVD risks were detected (*p* > 0.05).

In the sensitivity analyses, the estimates did not change appreciably after adjusting for additional covariates, excluding participants with frequent use of sleep aid medications, or excluding participants developing or dying of CVDs during the first 2 years of follow-up (data not shown). However, the association between DDF and ischemic stroke incidence was no longer significant (HR 1.04, 95% CI 0.97–1.11) after additionally controlling for DIMS and EMA.

Sex-specific modeling showed that associations between DIMS, EMA, DDF, and total CVD incidence were consistent among male and female participants (table 3). However, as for specific types of CVD, the association between DDF and IHD incidence was significantly stronger in women than in men. A similar pattern was found for the association between EMA and acute MI incidence (*p* for interaction <0.05). Moreover, associations of DDF with the incidence of stroke and its subtypes were identified only in male participants, although the sex heterogeneity was not statistically significant (*p* for interaction >0.05).

**Table 3:**
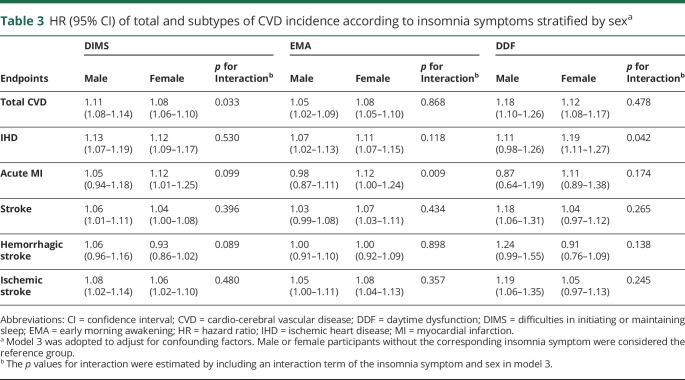
HR (95% CI) of total and subtypes of CVD incidence according to insomnia symptoms stratified by sex^a^

### Associations of any insomnia symptoms and number of insomnia symptoms with CVD incidence

Overall, 16.4% of participants reported having any insomnia symptoms, and the proportions of participants reporting 1, 2, and 3 insomnia symptoms were 10.0%, 5.2%, and 1.2%, respectively. After adjustment for potential confounders, having any insomnia symptoms was associated with increased risks of total CVD, IHD, and total and ischemic stroke incidence; the respective HRs (95% CIs) were 1.08 (1.07–1.10), 1.12 (1.09–1.15), 1.04 (1.02–1.07), and 1.06 (1.03–1.09).

**Figure 1:**
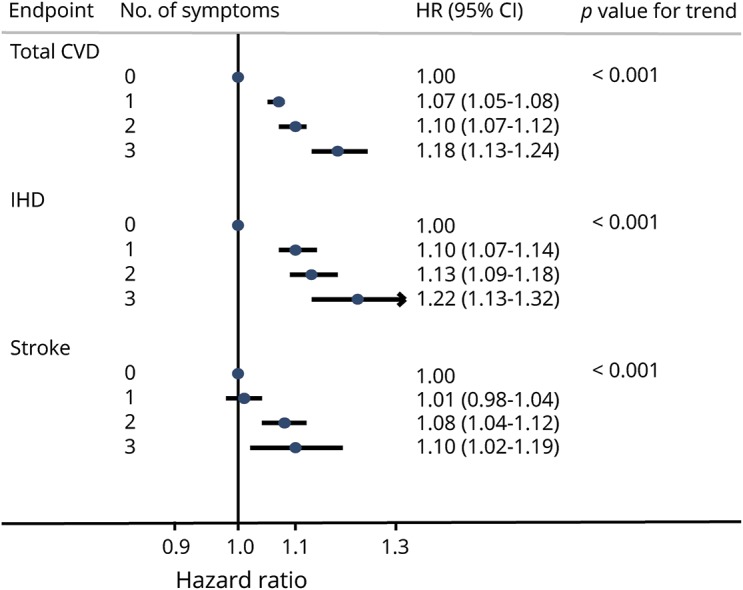
HR (95% CI) of total and subtypes of CVD incidence according to number of insomnia symptoms Models were adjusted for age; education level; annual household income; marital status; alcohol consumption; smoking status; tea consumption; physical activity level; intake frequencies of red meat, fresh fruits, and fresh vegetables; family history of heart attack and stroke; body mass index; prevalent hypertension and diabetes mellitus; frequent use of sleep aid medications; frequency of snoring; and depression or anxiety symptoms. Models were stratified by sex, 10 study areas, and baseline age in 5-year intervals. The *p* values for trend were estimated by modeling the number of insomnia symptoms as a continuous variable. CI = confidence interval; CVD = cardio-cerebral vascular disease; HR = hazard ratio; IHD = ischemic heart disease.

Compared with those without any insomnia symptoms, participants with 1, 2, or 3 symptoms had a 7%, 10%, or 18% higher risk of total CVD incidence, respectively (*p* < 0.05, figure 1). A larger number of insomnia symptoms was associated with increased risk of total CVD incidence (*p* for trend <0.001). Similar trends were also detected for incidences of IHD and total and ischemic strokes (*p* for trend <0.05).

### Subgroup analysis by baseline characteristics

**Figure 2:**
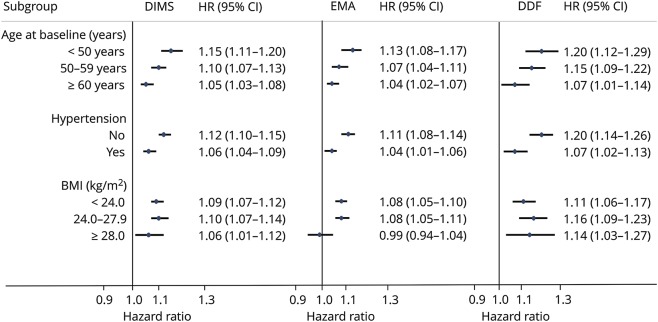
HR (95% CI) of total CVD incidence according to insomnia symptoms stratified by baseline characteristics All *p* values for interaction were <0.05 except for the interaction between daytime dysfunction (DDF) and body mass index (BMI). Model 3 was adopted to adjust for confounding factors. CI = confidence interval; CVD = cardio-cerebral vascular disease; DIMS = difficulties in initiating or maintaining sleep; EMA = early morning awakening; HR = hazard ratio.

Results of subgroup analysis indicated that baseline age, BMI, and prevalent hypertension modified the associations between insomnia symptoms and total CVD incidence (figure 2). The associations of DIMS, EMA, and DDF with total CVD incidence were consistently stronger in younger participants (*p* for interaction <0.05). Stronger associations for DIMS, EMA, and DDF were also identified in participants without hypertension at baseline (*p* for interaction <0.05). Compared with participants with obesity at baseline, normal-weight or overweight participants had higher increments of CVD risks associated with DIMS and EMA (*p* for interaction <0.05). No effect modification was identified by sleep duration, physical activity level, or prevalent diabetes mellitus at baseline (data not shown).

## Discussion

In this large-scale prospective study, we observed that the 3 insomnia symptoms of DIMS, EMA, and DDF were moderately associated with increased risks of total and specific types of CVD among Chinese adults. A positive dose-response relationship was identified between the number of insomnia symptoms and increased CVD risks. In addition, the associations between the 3 symptoms and CVD incidence were consistently more evident in younger adults or participants without hypertension at baseline.

Our study identified moderate associations between insomnia symptoms and total CVD incidence, which were consistent with findings from a number of previous cohort studies.^6,18^ A systematic review of 13 cohort studies with a combination of 122,501 participants indicated that insomnia disorder determined an increased risk of developing or dying of CVD (relative risk 1.45, 95% CI 1.29–1.62).^10^ However, the comparability across those studies was compromised by the inconsistency in the definitions of insomnia.^10^ A previous study of 23,447 US men investigated associations between individual insomnia symptoms and CVD mortality.^19^ The results showed that men with difficulty initiating sleep or nonrestorative sleep most of the time had a 55% or 32% increased risk of CVD mortality. Another study of 13,227 Swedish adults found that affirming moderate or considerable problems with at least 1 of 4 insomnia symptoms was associated with increased CVD incidence only in women (HR 1.4, 95% CI 1.2–1.6).^13^

As for risks of specific types of CVD, our results indicated that DIMS, EMA, and DDF were associated with increased risks of IHD incidence and total and ischemic stroke incidence. These results were in line with a systematic review that related insomnia to increased risks of CHD and stroke incidence (relative risk 1.28 and 1.55).^9^ Another cohort study of 52,610 Norwegian adults associated experiencing DIMS almost every night with an increase in acute MI incidence (HR 1.45 and 1.30).^20^ In our study, we also identified an association between DIMS and increased risk of acute MI incidence. Although a sufficient number of hemorrhagic stroke cases were observed during the follow-up time due to relatively high incidence rate among Asian population,^21,22^ we did not identify any associations between insomnia symptoms and hemorrhagic stroke incidence. This implied possible heterogeneity in the associations between insomnia and stroke subtypes, the underlying mechanisms of which warrant further research.

Sex heterogeneity was detected in the associations of insomnia symptoms with IHD and acute MI incidence. Similar to our results, the above-mentioned Norwegian cohort study also found that women had higher relative risks of acute MI associated with difficulties initiating sleep compared with men (*p* for heterogeneity = 0.009).^20^ Two Swedish cohort studies also found that difficulty maintaining sleep or any insomnia symptoms was associated with increased risks of cardiovascular events only in women.^13,23^ Such sex heterogeneity might be caused by different sleep characteristics or patterns between men and women^24^ or may be influenced by estrogen secretion in women.^25^

Our study is the first large-scale cohort study that identified positive dose-response relationships between the number of insomnia symptoms and risks of CVD, IHD, and stroke incidence. In contrast, a Finnish cohort study found no significant association between the number of insomnia symptoms and CHD incidence.^26^ The number of insomnia symptoms could reflect the severity of insomnia or complexity of underlying pathologies and thus was related to elevated CVD risks.

Results of subgroup analysis demonstrated that baseline age, BMI, and prevalent hypertension modified the associations between insomnia symptoms and total CVD incidence. In line with our study, a Swedish cohort study of 41,192 adults identified a significant interaction between age and difficulty falling asleep on CVD incidence.^23^ Another cohort study of 85,752 adults also indicated that insomniacs-to-noninsomniacs incidence rate ratio of stroke was highest among young adults.^7^ One possible explanation for the moderating effects of BMI and baseline hypertension is that the relationship between insomnia and future CVD risk could be mediated through developing obesity and hypertension during follow-up.^27,28^ Therefore, the associations were weakened among participants with preexisting obesity or hypertension at baseline. The findings of attenuated associations in older adults could be explained by relatively higher baseline CVD risks among elderly participants or the fact that a larger proportion of elderly participants had already developed intermediate diseases or disorders (e.g., obesity, hypertension, or diabetes mellitus) at baseline.

Several proposed biological mechanisms relate insomnia to CVD risks. Previous experimental and epidemiologic studies suggested that insomnia was related to elevated levels of inflammatory cytokines^29^ and sympathetic nervous activation^30^ and could lead to metabolic and endocrine disruptions.^31,32^ There was also evidence that sleep deprivation or poor sleep quality was related to levels of leptin and ghrelin, which could result in obesity and impaired glucose tolerance.^33^ In addition, several previous studies related insomnia to the pathogenesis and intermediate risk factors of CVD such as atherosclerosis and high blood pressure.^34,35^ Our findings of moderating effects of prevalent hypertension and obesity provided supporting evidence for these mechanisms.

Our results implied that preclinical insomnia symptoms could be considered modifiable risk factors for subsequent CVD incidence. At present, the clinical diagnostic criteria for insomnia are inconsistent,^16^ and there is a lack of widely accepted criteria for the classification of insomnia phenotypes.^36,37^ In contrast, individual insomnia symptoms are better defined and more feasible to assess with questionnaires in large-scale population studies and clinical practice. Moreover, it is reasonable that insomnia symptoms are more modifiable and precisely targetable through behavioral therapies before developing into clinically significant insomnia disorder.^38^ Therefore, future clinical trials or community-based intervention studies should be conducted to test whether lifestyle or sleep hygiene interventions for insomnia symptoms can reduce subsequent CVD risks.^8,39^ This study also implied that adults with multiple insomnia symptoms are at even higher risk of developing CVDs; this dose-response relationship should draw clinical attention. The moderating effect of age revealed that, although insomnia is more prevalent in older adults, it is indeed more detrimental for young adults in terms of CVD risks. In the present study, DDF was not associated with ischemic stroke incidence after controlling for DIMS and EMA, implying different physiologic impacts and health consequences of specific insomnia symptoms.^12^

This study is the first-ever large cohort study to examine the associations of individual insomnia symptoms with CVD incidence among male and female adults, with plenty of established and potential confounding factors being controlled. However, this study still has several limitations. First, we did not collect information on nonrestorative sleep, which is another common insomnia symptom,^1^ during the baseline survey, whereas other insomnia symptoms were well collected and well defined with quantitative criteria. Second, the validity of self-reported insomnia symptoms in this study has not been fully examined. Therefore, our findings need to be interpreted with caution due to possible information bias. However, the potential misclassification of baseline insomnia symptoms in this study was unlikely to depend on future CVD incidence and thus could only bias the results toward the null hypotheses. Furthermore, this study could still suffer from residual confounding bias due to uncollected covariates. For example, shift work and obstructive sleep apnea are established risk factors for CHD or stroke^40,41^ and could interfere with insomnia symptoms. Nevertheless, we did assess snoring symptom in the baseline survey and have adjusted for it in the main analysis. Finally, insomnia symptoms were assessed only once at baseline survey, so we were unable to examine the associations while taking into account the changes in symptoms over time. Future research will be conducted using repeatedly measured exposure data from the resurvey of CKB Study.

This large-scale cohort study demonstrated that individual insomnia symptoms are independent risk factors of total CVD, IHD, and ischemic stroke. The associations could be modified by age and prevalent hypertension at baseline. Therefore, early detection and intervention targeted at individual insomnia symptoms may have the potential to reduce subsequent CVD risks, especially among young adults and adults who have not developed hypertension.

## Glossary

BMI: body mass index
CHD: coronary heart disease
CI: confidence interval
CKB: China Kadoorie Biobank
CVD: cardio-cerebral vascular disease
DDF: daytime dysfunction
DIMS: difficulties in initiating or maintaining sleep
EMA: early morning awakening
HR: hazard ratio
ICD-10: *International Classification of Diseases, 10th Revision*
IHD: ischemic heart disease
MI: myocardial infarction
